# Cross-cultural adaptation and psychometric evaluation of the early childhood oral health impact scale (ECOHIS) in chilean population

**DOI:** 10.1186/s12955-018-1057-x

**Published:** 2018-12-16

**Authors:** Carlos Zaror, Claudia Atala-Acevedo, Gerardo Espinoza-Espinoza, Patricia Muñoz-Millán, Sergio Muñoz, María José Martínez-Zapata, Montse Ferrer

**Affiliations:** 1 Department of Pediatric Dentistry and Orthodontic, Faculty of Dentistry, Universidad de La Frontera, Manuel Montt, 112 Temuco, Chile; 2 Center for Research in Epidemiology, Economics and Oral Public Health (CIEESPO), Faculty of Dentistry, Universidad de La Frontera, Temuco, Chile; 3 Universitat Autònoma de Barcelona, Barcelona, Spain; 4 Department of Public Health, Faculty of Medicine, Universidad de La Frontera, Temuco, Chile; 5 Iberoamerican Cochrane Centre, Biomedical Research Institute Sant Pau (IIB Sant Pau), Barcelona, Spain; 6 CIBER Epidemiología y Salud Pública (CIBERESP), Madrid, Spain; 7 Health Services Research Group, IMIM (Hospital del Mar Medical Research Institute), Doctor Aiguader, 88, 08003 Barcelona, Spain

**Keywords:** Oral health, Quality of life, Questionnaires, Psychometrics, Outcome assessment, Child

## Abstract

**Background:**

The Early Childhood Oral Health Impact Scale (ECOHIS) measures the impact of dental diseases on Oral Health-Related Quality of Life both in children and their families. The aim of this study was to develop a Chilean Spanish version of the ECOHIS that is conceptually equivalent to the original and to assess its acceptability, reliability and validity in the preschool population of Chile.

**Methods:**

The Chilean version of the ECOHIS was obtained through a process including forward and back-translation, expert panel, and cognitive debriefing interviews. To assess metric properties, a cross-sectional study was carried out in Carahue, Southern Chile (April–October 2016). Children younger than six years old without systemic diseases, disabilities or chronic medication from eleven public preschools were included. Parents were invited to complete the Chilean version of the ECOHIS, PedsQL™4.0 Generic Core and PedsQL Oral Health scales, and to answer global questions about their children’s general and oral health. A subsample was administrated ECOHIS a second time 14–21 days after. A clinical examination was performed to assess dental caries, malocclusion, and traumatic dental injuries. Reliability was evaluated using measures of internal consistency (Cronbach’s alpha) and reproducibility (Intraclass correlation coefficient - ICC). Construct validity was assessed by testing hypotheses based on available evidence about known groups and relationships between different instruments.

**Results:**

The content comparison of the back-translation with the original ECOHIS showed that all items except one were conceptually and linguistically equivalent. The cognitive debriefing showed a suitable understanding of the Chilean version by the parents. In the total sample (*n* = 302), the ECOHIS total score median was 1 (IQR 6), floor effect was 41.6%, and ceiling effect 0%. Cronbach’s alpha was 0.89 and the ICC was 0.84. The correlation between ECOHIS and PedsQL™4.0 Generic Core was weak (*r* = 0.21), while it was strong-moderate (*r* = 0.64) with the PedsQL Oral Health scale. In the known groups comparison, the ECOHIS total score was statistically higher in children with poor than excellent/very good oral health (median 11.6 vs 0, *p* < 0.01), and in the high severity than in the caries-free group (median 8 vs 0.5, *p* < 0.01). No differences were found according to malocclusion and traumatic dental injuries groups.

**Conclusions:**

These results supported the feasibility, reliability and validity of the Chilean version of ECOHIS questionnaire for preschool children through proxy.

## Background

Oral diseases are highly prevalent in children worldwide despite the improvement in oral health indices initiated in the last decades [1–3]. It is well known that their consequences on children are serious and can affect their quality of life [4–8]. Early childhood caries continues to be a serious public health problem in Chile, with a prevalence that can reach 80% at 4 years of age [9–11]. Oral Health-Related Quality of Life (OHRQoL), together with clinical indicators, can jointly provide a more comprehensive assessment of the patient’s oral health [12]. The OHRQoL has been defined as a multidimensional concept which includes a subjective evaluation of the individual’s oral health, functional well-being, expectations and satisfaction with care, and their sense of self [12].

The knowledge of the OHRQoL might help to improve the development of effective oral health programs and services because it permits the assessment of young children’s perceived needs, and treatment strategy effectiveness [13]. This can contribute to the identification of groups with a higher level of need, to prioritize public health programs for care of children and adolescents, and to improve access to care [14]. The use of OHRQoL as an outcome measure is consistent with patient-centered care, being crucial in understanding the effectiveness of treatment from the patients’ perspective [12].

Several instruments have been developed to assess the OHRQoL, yet few of them have been specifically designed for preschoolers. The first OHRQoL questionnaire for this age group was the Michigan Oral Health-Related Quality of Life (Michigan OHRQoL) in 2003 [15]. Subsequently, the Early Childhood Oral Health Impact Scale (ECOHIS) was developed in 2007 [16], the Pediatric Oral Health-Related Quality of Life (POQL) in 2011 [17] and the Scale of Oral Health Outcomes for 5-year-olds (SOHO-5) in 2012 [18]. POQL and the ECOHIS measure the OHRQoL impact of dental diseases not only on the children, but also on their families. It is important because oral health conditions have an indirect impact on parents and family members, because they result in lost workdays or in having to spend time and money on dental care [19, 20]. The ECOHIS demonstrated high reliability [21, 22], good validity [23, 24] and responsiveness [25, 26], and it has been adapted into about 10 languages and countries [21–24, 27–31], including Spanish for Argentina [32].

Culture is an important factor that can influence a person’s activities, thinking and behavior. As countries differ regarding public health strategies, attitudes, socioeconomic conditions and other factors, the expression of their culture can change between populations [33], and instruments to measure Health Related Quality of life (HRQoL) should go through a cultural adaptation process before being used in a different country. Therefore, even among Spanish speaking countries it is usual to develop country-specific versions of instruments measuring HRQoL [34–36]. Even when the translation is performed with great precision, cultural factors may not be accurately conveyed. In order to study the health care needs of people with diverse cultural backgrounds, research instruments must be reliable and valid in each culture studied [37].

The aim of this study was to develop a Chilean Spanish version of the Early Childhood Oral Health Impact Scale (ECOHIS) that is conceptually equivalent to the original and to assess the acceptability, reliability and validity of this version in the preschool population of Chile.

## Methods

The study was performed in two phases. In the first phase, the scale was translated into Spanish and adapted to the Chilean culture. In the second phase, the psychometric properties were tested among a sample of parents of preschool children. The Ethics Committee of the Universidad de La Frontera approved the study protocol (resolution n° 061/2015).

### Early childhood Oral Health impact scale (ECOHIS)

The ECOHIS is a proxy-reported questionnaire developed in USA for measuring the OHRQoL of preschool children and their families [16]. It comprises of 13 items, covering six domains in two sections. The child’s impacts section contains 4 domains: symptom (1 item), function (4 items), psychology (2 items) and self-image and social interaction (2 items). The family’s impacts section contains 2 domains: parental distress (2 items) and family function (2 items). Response categories for each question are rated on a 5-point Likert scale to record how often an event has occurred during the child’s life: 0 = never, 1 = hardly ever, 2 = occasionally, 3 = often, 4 = very often, and 5 = don’t know. ECOHIS scores are calculated as a simple sum of the response codes for the child and family sections separately and also a total score, after recoding all “Don’t know” responses as “missing”. In cases with up to 2 missing responses in the child section or 1 missing response in the parent section, they were ascribed the average score of the rest of the items for that section. Parents missing responses to more than two child items and one family item were excluded from the analysis. Thus, the total score ranges between 0 and 52, with higher scores indicating a greater impact of oral problems and therefore worse OHRQoL [16].

### Linguistic and cultural adaptation

Standard methods were used to translate and culturally adapt the instrument [38, 39]. The Spanish translation of the ECOHIS was carried out independently by two professional linguists, both native Chilean Spanish speakers, with a high level of fluency in English. The focus of these forward translations was achieving a conceptual, rather than literal, equivalence. In addition each translator scored the difficulty in finding the conceptual equivalence in translation of each of the items from 1 (least difficulty) to 10 (maximum difficulty). To obtain a first consensual version, a joint revision of the two Chilean Spanish translations was undertaken by a panel composed of two experts in OHRQoL assessment, two pediatric dentists and the two translators. Then this first Chilean version was reviewed by a panel of parents of pre-school children (3 fathers and 4 mothers) to check its understanding and clarity. This pre-final version was translated back into English by two native American-English speakers. The difficulty in finding the linguistic equivalence in back-translation was also evaluated by translators. The equivalence between the original version and back-translation was evaluated by the expert panel who rated the items as: A (conceptually and linguistically equivalent to the original item), B (functionally equivalent, but with grammatical differences), or C (equivalence is not obvious). The report on equivalence between original and back-translated versions was sent to the authors of the original ECOHIS for evaluation.

As a last step, cognitive debriefing interviews were carried out on 15 parents (2 fathers and 13 mothers, aged 24 to 37 years old) of children between 2 and 5 years of age to evaluate the understandability and clarity of this preliminary version. Cognitive debriefing interviews included: first, asking parents to complete the questionnaire independently; and second, performing additional open questions in an effort to assess the content of the adaptation. This technique allowed assessing what the parents understood in the adapted version. For this purpose, we developed a set of questions to be used during the interview to obtain standardized information, such as: “In your own words, what do you think this question is asking? What does this item mean to you?” (Supplementary data). We recorded the conversations and took notes during the cognitive interviews. Then, we transcribed the audiotapes to prepare an item-by-item summary of each section of the questionnaire and modification recommendations if necessary.

### Study of the metric properties

A cross-sectional study was carried out in the city of Carahue, Southern Chile, from April 2016 through October 2016 to test the psychometric properties of the Chilean version of ECOHIS. Eleven public preschools were included, which are funded by the Chilean government for children younger than six years old.

Two- to 5-year-old children without any systemic diseases, disabilities or chronic medication were included. A written consent from the parents was obtained and the children gave their verbal consent for considering their participation in the study. The parents were invited to a meeting in the school, during which a dental examination of the participating children was performed and parents were asked to self-complete three questionnaires on their child: one measuring general HRQoL (PedsQL™4.0 Generic Core scale for toddlers), and two on OHRQoL (ECOHIS and PedsQL Oral Health). In addition, the parents completed a structured questionnaire to compile information on the child’s age, gender, socioeconomic status, history of oral hygiene habits, as well as their overall and dental health status. We sent by regular mail the questionnaires to parents who did not attend the meeting.

Three experienced researchers performed the dental examinations in the classroom. After cleaning the tooth surfaces with a toothbrush, a visual inspection of the oral cavity was performed under artificial light. The examiners were blinded to the questionnaire responses. The diagnosis of caries was based on the criteria proposed by the World Health Organization in the Oral Health Survey Basic Methods for Epidemiological Studies [40]. The types of traumatic dental injury were classified according to Andreasen & Andreasen [41] and the malocclusion was assessed according to the presence or absence of at least one of the following: anterior open bite, overjet > 4 mm and anterior cross-over bite [4].

Prior to beginning the study, the researchers were trained in dental examination to increase the degree of inter-examiner agreement. The training consisted of a stage in which the examination teams, each composed of an examiner and a recorder, received theoretical training on the study protocol and diagnostic criteria, as well as on how to complete a clinical record and a systematic dental examination. A group of 15 children were then examined to test the inter-examiner agreement on caries and malocclusions traits, with kappa coefficients of 0.83 and 0.70, respectively. A series of 20 pictures were used to assess reliability on traumatic dental injury (kappa = 0.79).

### Sample size

According to sample size recommendations to assess construct validity, ceiling/floor effects, internal consistency and factorial analysis, 2 to 20 participants per item are required, with an absolute minimum of 100 to 250 subjects [42–44]. Considering that the highest number of participants recommended per item is 20, and assuming a 15% of potential missing answers, the sample size required was of 300 children.

### Statistical analysis

A descriptive analysis of the sociodemographic characteristics and the results of the oral examination was performed. Mean, standard deviations, score range, and percentage of patients with the lowest (floor effect) and highest theoretical scores (ceiling effect) were calculated in order to examine the scores’ distribution of the ECOHIS. Reliability was assessed following two approaches: internal consistency was evaluated using Cronbach’s alpha; and test-retest reproducibility was assessed using the intraclass correlation coefficient (ICC) calculated by two-way random effects analysis of variance. Test-retest subsample was selected by randomization of 50% of the participants at each school, who received the questionnaires by mail 2–4 weeks after the school meeting. Parents who reported change in their child’s oral health status were excluded of this analysis.

Confirmatory Factor Analysis (CFA) was performed to assess the measurement model of the ECOHIS. To test the structure in two sections proposed by developers of ECOHIS (Child and Family impact sections), as well as for the existence of a general factor (the ECOHIS total score), a 2nd order model structure was imposed in the CFA. The CFA was performed using the robust unweighted least squares (ULSMV), and its goodness of fit was assessed using the Confirmatory Fit Index (CFI) and the Tucker-Lewis Index (TLI), which should be above 0.95, and the Root Mean Square Error of Approximation (RMSEA), which indicates an adequate fit below 0.08. The CFA was conducted with MPlus 5 [45].

Construct validity evaluation was based on known groups defined by results of dental examination (caries, traumatic dental injuries and malocclusion) and by responses from the parents about the child’s overall and dental health with a 5-Likert scale (Excellent, Very Good, Good, Fair, and Poor). We hypothesized worse child OHRQL (higher scores on the two sections of the ECOHIS) among children with some dental disease identified in the dental examination and among those whose overall and dental health was rated as fair or poor. Given the clearly skewed distribution of the ECOHIS score, we decided to use nonparametric analysis and Kruskal-Wallis or Mann Whitney tests were used to assess ECOHIS differences among these groups. To quantify the magnitude of the difference, effect size was calculated as the difference between means divided by the standard deviation pooled from the two groups. Effect sizes of 0.2, 0.5 and 0.8 were defined as small, moderate and large, respectively [46].

Additionally, to examine convergent and discriminant validity, correlations of ECOHIS scores with the PedsQL™4.0 Generic Core and PedsQL Oral Health scales were calculated using Spearman correlation coefficients, interpreted as follows: negligible relationship when r is < 0.20; weak when 0.20–0.40; moderate when 0.40–0.60; strong-moderate when 0.60–0.80; and strong relationship when > 0.80 [47]. Convergent validity involves demonstrating that different instruments measuring a similar concept inter-correlate at least moderately. We hypothesized moderate to strong correlation coefficients between ECOHIS and PedsQL Oral Health, since both were designed to measure OHRQoL. In contrast, discriminant validity is the extent to which a measure does not correlate too strongly with those measures intended to assess different traits. Therefore, we hypothesized that correlations between ECOHIS and PedsQL™4.0 Generic Core is low, due to differences between OHRQoL and HRQoL. The data analyses were performed using Stata 13 (Stata Corp, College Station, TX, USA).

## Results

### Cross-cultural adaptation process

**Fig. 1:**
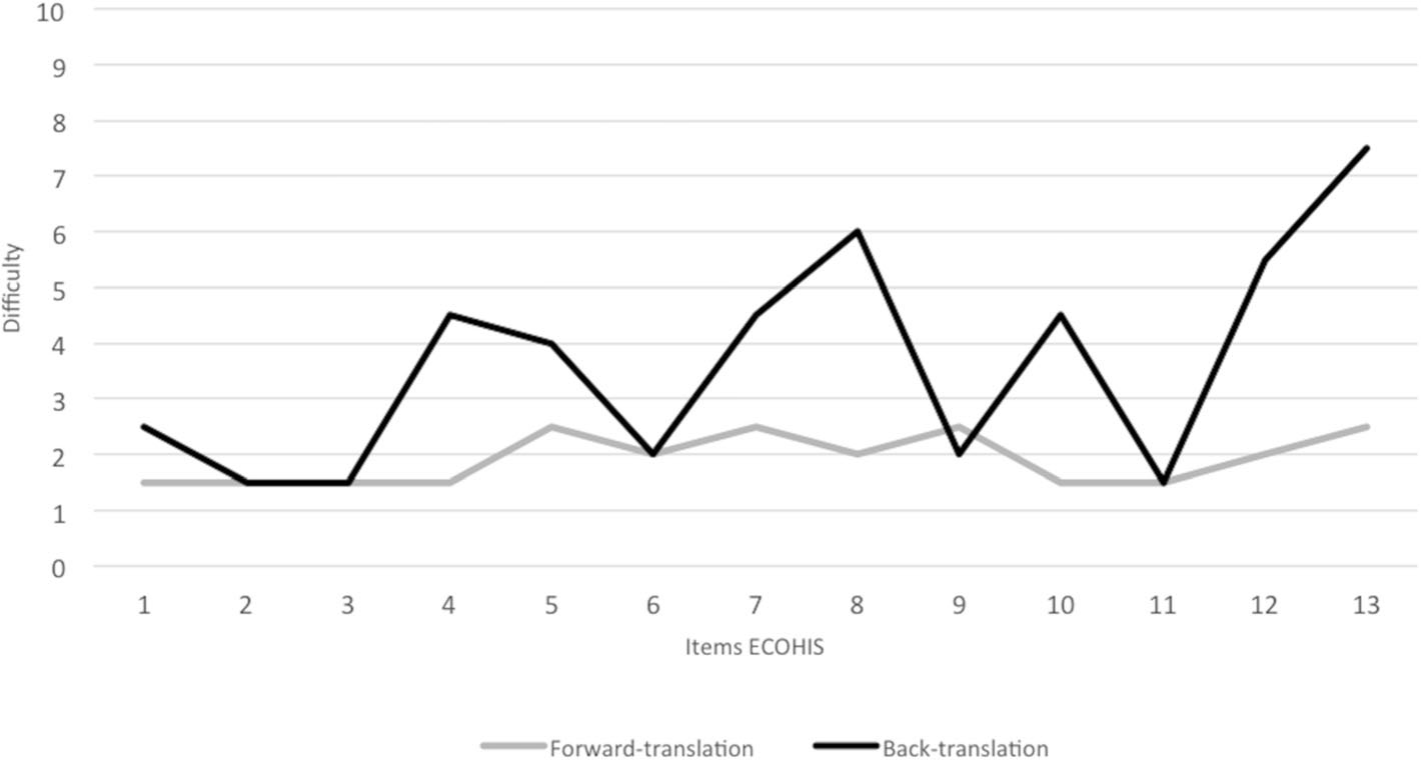
Average difficulty to find the conceptual equivalence, as reported by translators in the forward and back-translations

The average difficulty for the forward translation of the items into Chilean Spanish was < 2.5. Regarding the back-translation, the average difficulty was of 7.5 for item 13, 6 for item 8, 5.5 for item 12 and below or equal to 4.5 for the rest (Fig. 1). For content comparison between back-translation and the original version, the expert panel rated all items as A (conceptually and linguistically equivalent), except item 13 which was rated as C (equivalence is not obvious). This was due to the replacement of the term “financial impact” by “important economic cost” after members of the panel of pre-school children parents claimed they did not understand the first expression. The author of the original ECOHIS reviewed the Spanish and the English back-translated versions without identifying any lack of equivalence regarding the original.

Finally, the cognitive debriefing showed that the instructions, items and response choice were easy to understand by parents. The parents thought about the whole vital cycle of their child when answering the questions. Some parents had difficulty defining in their own words the terms “frustrated” and “irritable”, however they were able to differentiate between them. None of the parents had problems to differentiate among the different response options. All parents agreed that the questions are intended to evaluate OHRQoL. No modification was necessary as a result of the cognitive debriefing interviews.

### Psychometric study

The population of Carahue preschools included a total of 435 children, two of them were excluded for presenting special health care needs, twelve because their parents did not sign the informed consent, and 93 children were absent at the time of dental examination. Of the 328 parents included, 26 did not return the questionnaires (response rate = 92.1%). In total, 302 children were fully evaluated (Table 1), comprising 163 boys and 139 girls, with an average age of 4.0 (SD = 1.1) years. Most were of low socioeconomic status, 40.9% of the parents reported that their children have good general health and 36.5% good oral health. The prevalence of dental caries, malocclusion and traumatic dental injuries was 53.6, 39.4 and 14.5% respectively.

**Table 1:** Demographic and clinical characteristics of the children assessed in the study

Variables	*n* (%)
Child’s age in years (mean ± SD)	4.0 (1.1)
Child’s gender	
Male	163 (54.0)
Female	139 (46.0)
Socioeconomic status	
Low	229 (75.8)
Medium-high	73 (24.2)
Child’s general health, reported by parents	
Excellent	43 (14.3)
Very good	86 (28.5)
Good	123 (40.9)
Regular	49 (16.3)
Poor	–
Child’s oral health, reported by parents	
Excellent	44 (14.6)
Very good	59 (19.6)
Good	110 (36.5)
Regular	70 (23.3)
Poor	18 (6.0)
Tooth brushing	
Once a day or less	51 (16.9)
Twice or more	251 (83.1)
Simplified Oral Hygiene Index	
Good	19 (6.3)
Regular	223 (73.8)
Poor	60 (19.9)
Decayed, missing and filled teeth index (mean ± SD)	2,52 (SD 3.71)
Dental Caries	
Caries free (dmft = 0)	140 (46.3)
Low severity (dmft = 1–5)	108 (35.8)
High severity (dmft > 6)	54 (17.9)
Malocclusion	
Absence	183 (60.6)
Presence	119 (39.4)
Traumatic Dental Injuries	
None	258 (85.4)
Infraction	27 (8.9)
Enamel fracture	4 (1.3)
Avulsion	2 (0.7)
Discoloration	11 (3.6)

Table 2 shows the children’s parents extreme ECOHIS responses and reliability coefficients. All items were rated as “never” by over 60% of parents. The two items most frequently rated as “never” were in the child section: “avoided smiling or laughing” (92.4%) and “avoided talking” (93.7%). The two items most frequently rated as “very often” were in the family section, parents or family members having “been upset” (1.9%) and “feel guilty” (3.6%). The Cronbach’s alpha coefficient was 0.89 for the total score showing a good correlation within items. Among the subsample of 84 parents who completed the ECOHIS twice, the Intraclass Correlation Coefficient was 0.84 for the total score. Both reliability coefficients were above the recommended standard of 0.7 in the child and the family sections.

**Table 2:** ECOHIS extreme responses of children’s parents and reliability coefficients (*n* = 302)

Impacts	Never		Very often		Cronbach’s alpha (ICC)^a^
	n	%	n	%	
CHILD IMPACTS					0.88 (0.81)
How often has your child had pain in the teeth, mouth or jaws	188	62.3	3	0.9	0.86
How often has your child because of dental problems or dental treatments?					
Had difficulty drinking hot or cold beverages	223	73.8	1	0.3	0.85
Had difficulty eating some foods	216	71.5	1	0.3	0.85
Had difficulty pronouncing any words	240	79.5	1	0.3	0.87
Missed preschool, day care or school	252	83.4	–	–	0.86
Had trouble sleeping	263	87.1	–	–	0.86
Been irritable or frustrated	240	79.5	1	0.3	0.86
Avoided smiling or laughing	279	92.4	–	–	0.87
Avoided talking	283	93.7	–	–	0.88
FAMILY IMPACTS					
How often have you or another family memb because of your child’s dental problems or treatment?					0.80 (0.75)
Been upset	224	74.2	6	1.9	0.71
Felt guilty	214	70.9	11	3.6	0.74
Taken time off from work	248	82.4	1	0.3	0.76
How often has your child had dental problems or dental treatments that had a financial impact on your family?	252	83.7	3	1.0	0.77

^a^*ICC* Intraclass Correlation Coefficient

**Fig. 2:**
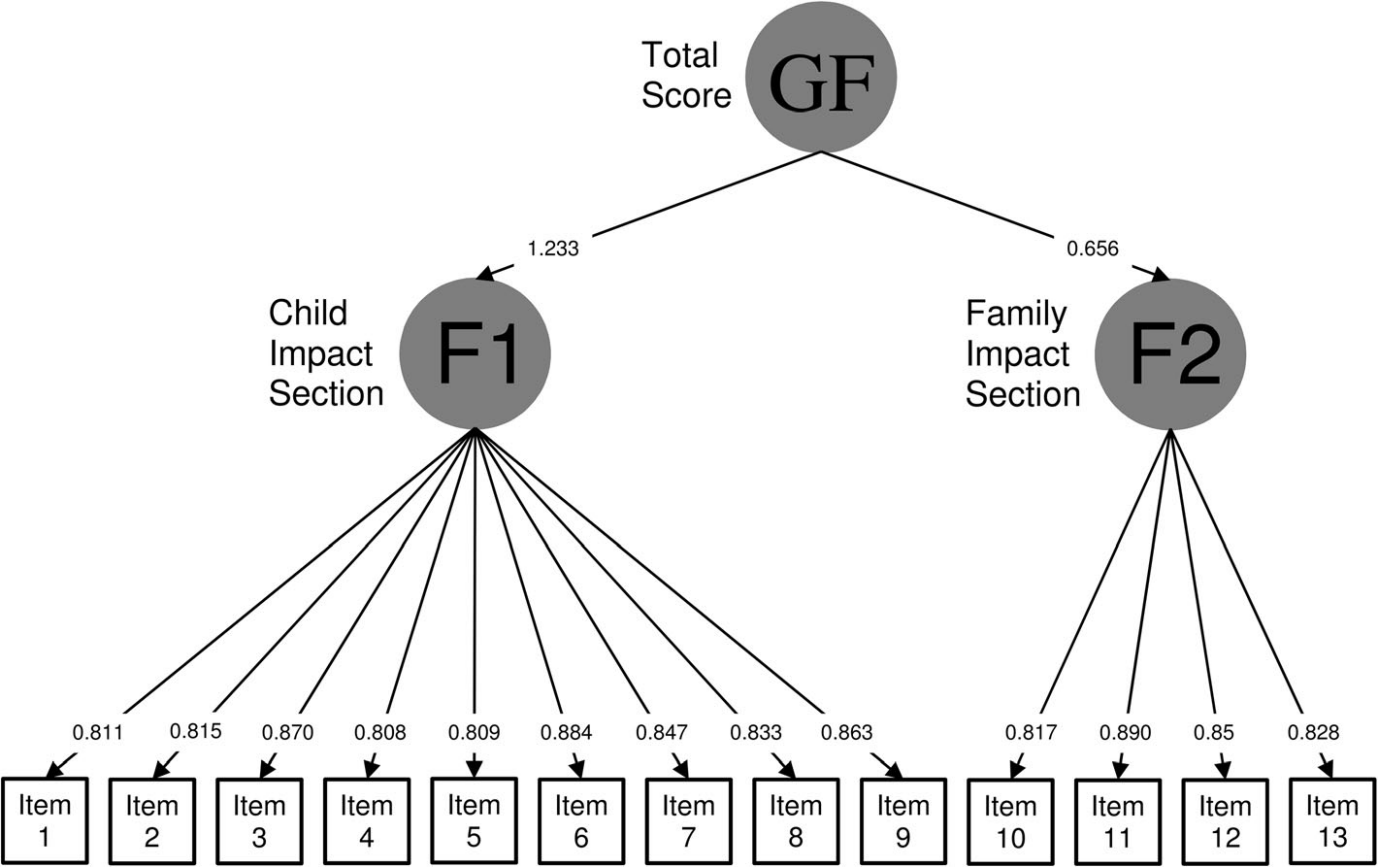
Confirmatory factor analysis to assess the measurement model of the ECOHIS

The measurement model consisted of two specific factors and a general factor (Fig. 2). Factor 1 includes the nine items composing the Child Impact Section; factor 2 includes the 4 items composing the Family Impact Section; and the latent construct for the total score includes both factors (Child and Family Impact). This CFA model presented excellent goodness of fit coefficients: CFI = 0.978, TLI = 0.988 and RMSEA = 0.065.

Distributions of the ECOHIS scores are presented in Table 3. The median of the total ECOHIS score was 1 (IQR 6), for child impact it was 1 (IQR 3) and 0 (IQR 2) for the family impact section. In the child impact section, 5.0% of the parents answered “Don’t Know” in at least one item and 1.7% in the family impact section. The floor effect was 41.6% and ceiling effect was negligible for the total score.

**Table 3:** Descriptive data of the distribution of the ECOHIS scores (*n* = 302)

Section/Scale	Number of items	Observed range	Median (IQR)	Mean (SD)	Percentage (%) of patients with				
					Any missing item	Any ‘Don’t Know’	Missing score	Floor effect	Ceiling effect
CHILD IMPACT SECTION	9	0–22	1 (3)	2.53 (4.07)	0.0	5.0	0.3	49.3	0.0
Symptom	1	0–4	0 (1)	0.58 (0.89)	0.0	0.7	–	62.9	1.0
Function	4	0–12	0 (2)	1.32 (2.24)	0.0	4.3	–	60.6	0.0
Psychological	2	0–6	0 (0)	0.48 (1.09)	0.0	0.3	–	77.8	0.0
Social	2	0–3	0 (0)	0.15 (0.52)	0.0	1.0	–	91.7	0.0
FAMILY IMPACT SECTION	4	0–14	0 (2)	1.5 (2.65)	0.0	1.7	0.3	61.3	0.0
Parental distress	2	0–8	0 (1)	1.01 (1.80)	0.0	1.7	–	66.2	1.7
Family function	2	0–6	0 (0)	0.51 (1.17)	0.3	1.0	–	78.8	0.0
ECOHIS TOTAL SCORE	13	0–31	1 (6)	4.04 (6.09)	0.3	6.3	0.3	41.6	0.0

***Floor effect*** percentage of patients with score = 0, ***Ceiling effect*** percentage of patients with maximum score (52)

Table 4 shows the results of the construct validity of ECOHIS based on known groups. As the child’s general health and oral health was rated worse by parents, the ECOHIS median total score was higher, but differences among groups were only statistically significant for oral health: from 0 when excellent/very good to 11.6 when poor (*p* < 0.01). Finally, regarding dental diseases, ECOHIS scores presented statistically significant differences among groups defined by dental caries (median 0.5, 2, and 8, p < 0.01), but differences between presence or absence of malocclusion or type of traumatic dental injuries were not significant. Effect sizes indicate large differences between groups defined by child’s oral health and dental caries.

**Table 4:** Construct validity of ECOHIS total score based on known groups (*n* = 298)

Variables	n	Median (IQR)	Mean (SD)	p	Effect size
Child’s general health reported by parents					
Excellent/Very good	130	1 (5)	3.84 (6.40)	0.13	
Good	119	2 (6)	3.92 (5.67)		0.01
Regular	49	3 (10)	6.34 (7.74)		0.38
Poor	–	–	–		
Child’s oral health reported by parents					
Excellent/Very good	104	0 (3)	1.83 (3.56)	< 0.01	
Good	109	1 (4)	2.70 (3.74)		0.24
Regular	67	6.1 (11)	7.89 (7.73)		1.09
Poor	18	11.6 (18)	14.51 (10.25)		2.50
Dental Caries					
Caries free (dmft = 0)	140	0.5 (3)		< 0.01	
Low severity (dmft = 1–5)	105	2 (5.1)			0.43
High severity (dmft > 6)	53	8 (13)			1.49
Malocclusion					
Absence	180	1 (5)	3.55 (5.48)	0.30	
Presence	118	2 (8)	4.85 (6.97)		0.22
Traumatic Dental Injuries					
Absence	255	1 (5)	3.99 (3.99)	0.11	
Presence	43	3 (7)	4.47 (4.47)		0.48

*dmft* Decayed, missing, and filled teeth index

Table 5 shows that the correlation of the total score of ECOHIS with the PedsQL™4.0 was strong-moderate with the Oral Health scale (*r* = 0.64), weak with the Generic Core scale (*r* = 0.21), and also when both scales were considered (*r* = 0.35). Finally, the correlation between the child and the family impact sections of ECOHIS was moderate (*r* = 0.57; *p* ≤ 0.001).

**Table 5:** Correlation of ECOHIS scores with PedsQL™4.0 Generic Core and PedsQL™4.0 Oral Health scales

	Early Childhood Oral Health Impact Scale (ECOHIS)		
PedsQL™	Child Impact Section	Family Impact Section	ECOHIS Total score
PedsQL™4.0 Generic Core scale	0.20*	0.16*	0.21*
PedsQL Oral Health scale	0.65*	0.51*	0.64*
PedsQL ™ 4.0 Generic Core and PedsQL Oral Health scales	0.35*	0.29*	0.35*

*Statistically significant at *p* < 0.001

## Discussion

We used a standard cross-cultural adaptation process to develop the Chilean version of the ECOHIS, which demonstrated good acceptability by parents; high reliability and good construct validity. The results are consistent with those obtained for the original ECOHIS and suggest that the Chilean version is conceptually and metrically equivalent.

“Don’t know” and/or missing responses may reflect comprehensibility problems [24]. In our sample, only one parent left some missing items and only 19 (6.3%) responded “Don’t know”, similarly to the original ECOHIS study (7%) [16]. However, other studies have shown higher “Don’t know” percentages [19–22]. The low percentage of “Don’t know” supports that the mode of administration (proxy-report) is not a limitation for the ECOHIS Chilean version. According to the ECOHIS proxy-report design [16], in our study most parents completed it during the school meeting, and those who did not attend it completed the questionnaire at home. No interview administration was needed, and no one required assistance to self-complete the questionnaire. Self-administration presents advantages, such as lower cost, preservation of participant’s anonymity, and reduction of interviewer bias [48]. Furthermore, studies with other OHRQoL instruments showed that administration mode (interview versus self-administered) does not influence the instruments’ scores [48–50]. On the other hand, evidence shows that parents underestimate the impact of children’s oral health problems, since they have a different perspective and limited knowledge, particularly related to social and emotional well-being [51]. Indeed, oral health problems directly observable by parents, such as physical complaints and functionality, concur better with children’s perceptions [52, 53]. However, in this age group due to their cognitive immaturity, limited social experience and continued dependency, parents are the best source of their child’s oral health [54]. As in the original version, we included parents with “Don’t know” responses in the analysis because a “Don’t know” response reflects an essential characteristic of the phenomenon under evaluation, rather than errors by the respondents [55].

The high floor effect observed in the total score (41.6%) and domain or section scores (ranging 49–92%) is congruent with the clinical characteristics of our participants, since over 40% of the sample was free of oral conditions. Although these results are similar to those obtained in other studies, which have also shown a strong floor effect for ECOHIS total score (ranging 20–54%) [16, 24, 29] they could indicate a limitation of the instrument. The ECOHIS Chilean version needs to be tested in a population with more oral problems to assess adequately the instrument’s more severe response levels. The ECOHIS has shown an excellent reliability, both in its internal consistency and its reproducibility, since its coefficient values were over 0.8 allowing to use its scores for the comparison between groups [56]. Our result of internal consistency for the child section (Cronbach’s alpha coefficient = 0.88) was similar to the 0.91 reported by the original English questionnaire, but it was lower for the family impact section (0.80 vs. 0.95). However, with exception of the original version, the family section usually shows a lower internal consistency (Cronbach’s alpha ranging 0.59–0.85) than the child impact section (ranging 0.74–0.92) [21, 22, 29], which may be due to the lower number of items rather than a lower consistency. In the test-retest reliability, the ICC for total score was the same as reported in the original version (0.84), but lower than reported in the French (0.95) [24] and Brazilian versions (ranging 0.94–0.99) [22, 57]. Despite this, the ICC value shows that the Chilean version of ECOHIS has an excellent test-retest reliability in which it is able to produce reproducible scores when it is administered at two different times [43].

The good results on equivalence with the original ECOHIS shown by its comparison with the back-translation of the Chilean-adapted version support the content validity of this new country version. The higher difficulty of the back-translation compared to the forward one, observed in our adaptation process, has been also described for other adapted instruments [58, 59]. As the first translation seeks conceptual equivalence, and the second one seeks a literal translation of the expressions, this back-translation can often be harder to carry out.

To the best of our knowledge, there is no previous publication describing the factor structure of the ECOHIS. Our results confirm the two- section structure proposed by the developers (child and family impact sections), as well as that correlations between them can be explained by the second order model representing the global OHRQoL. The confirmation of this measurement model in other country versions of the ECOHIS would be recommendable.

For construct validity, the Chilean version of the ECOHIS scale showed significant differences among groups defined by the children’s dental health status as reported by parents. These findings were consistent with previous studies where parents who perceived their child’s oral health as poor had significantly higher mean ECOHIS scores [16, 21, 24, 27]. Our results showed higher ECOHIS scores among those with more than 6 decayed teeth, compared to those who had 1–5 decayed teeth or to those who were caries-free. The large effect size in children with poor oral health status reported by parents and who have high severity of caries supports the parents’ recognition of oral health problems when they become evident, or when it manifests in the form of pain [60]. However, the ECOHIS was not able to discriminate presence or absence of malocclusion or type of traumatic dental injuries. Although the ECOHIS was originally developed to assess the impact of dental caries, it has been widely used to evaluate several oral pathologies [4, 61], but only few studies have validated this application: Peker et al. only found a moderate correlation with gingival index [21], and Scarpelli et al. showed a statistically significant association with discolored upper anterior teeth [22]. This is important because the ECOHIS has been used to measure OHRQoL in patients with traumatic dental injuries or malocclusion, not detecting any impact on the children [4, 62]. Further research is needed to explore whether this absence of impact can be due to the inability of the instrument to discriminate between certain degrees of these pathologies.

The poor correlation between ECOHIS and PedsQL™4.0 Generic Core scale suggests that ECOHIS captures additional information, which is not covered by instruments measuring HRQoL. This is in line with results reported by Lee et al., showing that the ECOHIS is more sensitive than PedsQL™4.0 measuring the impact of oral problems on preschool children [63]. As expected, a high correlation was found with the Oral Health scale of PedsQL™4.0 because it also could be considered specific for measuring OHRQoL [64, 65]. The moderate correlation between the child and the family impact sections of the scale found in our sample (*r* = 0.57) was similar to results reported in previous studies ranging 0.36–0.68 [16, 21, 27]. The correlation in the original English questionnaire between both sections was the lowest (Spearman’s *r* = 0.36, *p* ≤ 0.001), and the Turkish version the strongest (Spearman’s *r* = 0.68, p ≤ 0.001). Although child and family sections assess different aspects of child’s OHRQoL, both sections are related with the underlying construct.

The main limitation of this study was the homogeneity of the sample studied, since only preschoolers from public schools were included. Nonetheless, our sample is representative of children between 2 and 5 years old attending public preschools, and these children are the main target of Oral Health Policies in Chile. Another limitation was that information regarding the parents, such as age, gender, and educational level, was not registered. Finally, the responsiveness was not assessed; therefore, future studies are necessary to evaluate the capacity of the ECOHIS Chilean version to detect changes over time in a clinical or public health intervention.

## Conclusions

The Chilean version of the Early Childhood Oral Health Impact Scale was valid and reliable for assessing the OHRQoL in preschool children through proxy. The comparison with the original U.S. version shows similar results in reliability and validity, suggesting that the cross-cultural adaptation method followed has yielded an equivalent Chilean version.

Researchers and clinicians now have at their disposal an OHRQoL instrument for use in Chilean preschool children to assess the impact of oral disorders on them and their families, and also to facilitate the identification of groups at a higher risk of dental health inequity to improve their access to oral health care services.

## Abbreviations

dmft: Decayed, missing and filled teeth index
ECOHIS: Early Childhood Oral Health Impact Scale
HRQoL: Health-Related Quality of Life
ICC: Intraclass correlation coefficient
OHRQoL: Oral Health-Related Quality of Life
PedsQL™4.0: Pediatric Quality of life Generic Core scale
